# Impact of *Fusobacterium nucleatum* on immune cell interactions and gene expression in colorectal cancer: a retrospective cohort study

**DOI:** 10.3389/fimmu.2025.1629014

**Published:** 2025-09-11

**Authors:** Ronald Heregger, Florian Huemer, Richard Greil, Marina Canadas-Ortega, Gernot Posselt, Marieke E Ijsselsteijn, Christina Plattner, Dietmar Rieder, Zlatko Trajanoski, Anne Krogsdam, Eckhard Klieser, Daniel Neureiter, Silja Wessler, Lukas Weiss

**Affiliations:** ^1^ Department of Internal Medicine III with Hematology, Medical Oncology, Hemostaseology, Infectiology and Rheumatology, Oncologic Center, Paracelsus Medical University, Salzburg, Austria; ^2^ Austrian Breast and Colorectal Cancer Study Group (ABCSG), Vienna, Austria; ^3^ Cancer Cluster Salzburg, Salzburg, Austria; ^4^ Department of Biosciences and Medical Biology, Division of Microbial Infection and Cancer, Paris-Lodron University of Salzburg, Salzburg, Austria; ^5^ Center for Tumor Biology and Immunology (CTBI), Paris-Lodron University of Salzburg, Salzburg, Austria; ^6^ Department of Pathology, Leiden University Medical Center, Leiden, Netherlands; ^7^ Institute of Bioinformatics, Medical University of Innsbruck, Innsbruck, Austria; ^8^ Institute of Pathology, Paracelsus Medical University, Salzburg, Austria

**Keywords:** colorectal cancer, microbiota, *Fusobacterium nucleatum*, tumor microenvironment, RNA-Seq, immunosuppressive environment, immune cell interactions

## Abstract

**Background:**

Colorectal cancer (CRC) is a major global health concern. The presence of *Fusobacterium nucleatum* (Fn) in CRC can promote cancer progression by modulating the immune response and creating an immunosuppressive environment.

**Methods:**

A cohort of 107 patients with localized CRC treated between 2005 and 2017 was analyzed, categorizing tumors as Fn-positive (Fn^+^) or Fn-negative (Fn^−^) using quantitative PCR. Patient characteristics, tumor characteristics and survival data were compared between groups. We further performed bulk RNA sequencing and gene set enrichment analysis to explore differential gene expression between Fn^+^ and Fn^−^ CRC. Spatial immune cell interactions within the tumor microenvironment were characterized using imaging mass cytometry (IMC) and quantified through Voronoi tessellation-derived mixing scores.

**Results:**

In 45 out of 107 patients (42%) tumors were classified as Fn^+^. Fn positivity was significantly associated with poor tumor differentiation (p=0.008) but did not significantly impact overall survival (OS; log-rank p = 0.099) or disease-free survival (DFS, log-rank p=0.595). Fn^+^ tumors exhibited distinct immunological features: RNA sequencing identified significant downregulation of pathways involved in immune activation and antibacterial defenses. IMC demonstrated increased intratumoral interactions between immune cells, antigen-presenting cells, and tumor cells in Fn^+^ tumors compared to Fn^−^ tumors, though these differences were not observed at tumor margins. Furthermore, Fn persistence was confirmed in metastatic lesions, suggesting a potential role in tumor spread and disease progression.

**Discussion:**

Our findings suggest that Fn contributes to an immunosuppressive microenvironment in CRC, diminishing both antibacterial defense and anti-tumor immunity. Selective elimination of Fn may enhance treatment efficacy and warrants further investigation.

## 1 Introduction

Colorectal cancer (CRC) is a major global health concern, ranking as the third most frequently diagnosed cancer and the second most common cause of cancer-related mortality in 2024 (1). Besides age, the risk factors for CRC include both environmental and inherited components. Genetic conditions such as Lynch syndrome or familial adenomatous polyposis account for a minority of cases (2), highlighting the influence of environmental factors such as diet, lack of exercise, smoking, obesity, alcohol consumption, and antibiotic use (3, 4). These factors can alter the gut microbiota, fostering a dysbiotic environment enriched in pathogens such as *E. coli*, *Salmonella species*, and *Fusobacterium nucleatum* (Fn).

Fn is a gram-negative, rod-shaped anaerobe commonly found in the oral cavity and gastrointestinal tract, and is associated with periodontitis (5). Fn is a facultative intracellular bacterium, meaning it primarily exists extracellularly but can invade host cells under certain conditions. By evading immune responses and inhibiting apoptosis, Fn may persist within the tumor microenvironment, contributing to chronic inflammation and cancer progression (6).

Multiple studies have reported elevated Fn levels in stool samples of patients with colorectal adenomas (7, 8). Additionally, the amount of intracellular Fn can vary among different types of colorectal polyps, such as hyperplastic, sessile, tubular, and villous/tubulovillous, but the results differ among current studies (8, 9). Therefore, it was not surprising to see results indicating a higher prevalence of Fn in the fecal microbiota of CRC patients (7, 10, 11). The primary niche of Fn is the oral cavity, from which it can reach colorectal cancer cells via bacteremia (for instance, during dental treatment), or through the orthograde colonization of the gut and subsequent invasion of tumor cells via a compromised mucosal barrier (7, 11).

The correlation between stool and tissue Fn levels is inconsistent (8). Fn abundance tends to increase with CRC stage and is more prevalent in right-sided tumors, which are often characterized by microsatellite instability-high/mismatch repair deficient (MSI/dMMR), CpG island methylator phenotype (CIMP), and BRAF (11–16) mutations, suggesting a crucial role of Fn in the development and progression of CRC (17). While, Fn appears to be more relevant in the later stages of cancer progression than in the early oncogenesis of CRC (8), claims of intracellular localization should be interpreted with caution. Such evidence requires validation through techniques like FISH or electron microscopy; among the cited studies, only Li et al. provided such confirmation using FISH (13).

Fn has been proposed to promote tumorigenesis by inducing pro-inflammatory cytokines (e.g., IL-8, CXCL1) (18) recruiting immunosuppressive cells, and upregulating PD-L1 expression (6). These mechanisms contribute to immune evasion and a tumor-promoting microenvironment.

Given the significant role of Fn in the progression of CRC, it is crucial to delve deeper into its impact on CRC immunogenicity. In this retrospective cohort study, we aimed to investigate the role of Fn in CRC by assessing (i) Fn prevalence and clinicopathologic associations, (ii) analyze transcriptional differences in MSS/pMMR rectal cancers, (iii) quantify spatial immune interactions via imaging mass cytometry (IMC), and (iv) evaluate Fn persistence in metastases.

## 2 Materials and methods

This retrospective study gathered data from 107 patients with histologically confirmed localized CRC who were diagnosed and/or treated between 2005 and 2017 at the Department of Internal Medicine III of the Paracelsus Medical University Salzburg, Austria. Patients with synchronous metastases were excluded from this study. Of the 107 patients, 47 had rectal cancer and all were treated with neoadjuvant long-course chemoradiotherapy using capecitabine as a radiosensitizer. The remaining 60 patients with locally advanced colon cancer underwent primary surgery and received adjuvant chemotherapy with either capecitabine plus oxaliplatin, fluorouracil plus oxaliplatin, or capecitabine monotherapy, depending on the tumor stage, performance score, and age. The following data were extracted from medical records: 1) patient characteristics such as sex, age at CRC diagnosis, date of last follow-up, or death; 2) tumor characteristics such as primary tumor localization (right versus left), stage, histological grade, time point of metastasis detection, metastases distribution pattern, and predictive biomarkers (microsatellite/mismatch-repair status); and 3) (systemic) treatment.

### 2.1 Statistical analysis

STATA BE 18.0 was used for collecting and analyzing data. Statistical significance was set at P ≤ 0.05. Baseline characteristics were compared using cross-tabulation together with the chi-square test for categorical data. Continuous data were summarized using medians and ranges and compared between the groups using the Mann–Whitney test. Kaplan–Meier survival curves and log-rank tests were used to evaluate disease-free survival (DFS) and overall survival (OS) between the groups. DFS was calculated from the date of surgery until locoregional recurrence, the detection of distant metastases, or death. OS was calculated from the date of CRC diagnosis until death from any cause. Patients who were alive at the last contact were censored.

### 2.2 Pathological staining and analysis

Immunohistochemical analysis was conducted on 4-µm thick formalin-fixed paraffin-embedded (FFPE) sections. Each specimen was mounted on an adhesive glass slide and dried at 60°C for one hour. Standardized routine immunohistochemistry (IHC) protocols were used for deparaffinization, antigen retrieval, immunostaining, counterstaining, dehydration, coverslip application, and pretreatment in the immunohistochemical laboratory of the University Institute for Pathology of the Paracelsus Medical University Salzburg, Austria. Immunohistochemical staining for Fn was performed using a Ventana Benchmark Ultra instrument (Ventana Medical Systems, Tucson, AZ, USA; trademark of Hoffmann-La Roche AG, Basel, Switzerland) with anti-Fusobacterium antibody (Clone ABIN4888518, Diatheva S.R.L., Fano PU, Italy). Immunohistochemical staining for mismatch repair proteins was performed using a Dako Omnis Autostainer combined with the EnVision Plus System (Dako, Vienna, Austria) using anti-MLH1, -MSH2, -MSH6, and -PMS2 ready-to-use antibodies (MLH1: Clone ES05; MSH2: Clone FE11; MSH6: Clone EP49; PMS2: Cone EP51). All immunohistochemical staining procedures were performed by two experienced pathologists.

### 2.3 Molecular genetic analysis of Fn

The specimens used for molecular genetic analysis were obtained from diagnostic biopsies before tumor treatment.

Due to differences in tissue preservation and available material, Fn detection was performed using DNA-based qPCR in colon cancer samples and RNA-based qPCR in rectal cancer samples. To ensure assay robustness, consistent cycle conditions were applied, primer specificity was validated, and low-quality samples were excluded. Comparative analyses between Fn^+^ and Fn^−^ tumors were stratified by cohort to account for methodological differences.

#### 2.3.1 RNA-sequencing

Total RNA (isolated as described below) was submitted to the Medical University Innsbruck MultiOmics-Seq Core Facility (Innsbruck, Austria) for gene expression analyses. Libraries were generated using the QuantSeq 3’-mRNA Library Preparation kit (Lexogen GmbH, Vienna, Austria), following the manufacturer’s instructions with the following modifications to accommodate the variation in cross-linking effects in the FFPE-derived RNA. Denaturation at 85°C (step 2 in the protocol), altered cDNA size selection (step 16, 48µl PS), and altered library size selection (step 29, 30µl PB) were included. Further, the final amplification was performed in two steps, with an AMPure bead (Becton Dickinson Austria GmbH, Vienna, Austria) clean-up step in between, elution in 18µl of which 16µl was used for the second round of amplification, totalling 13 + 6 PCR cycles of amplification. The final libraries were multiplexed and sequenced using Illumina NovaSeq technology.

#### 2.3.2 Colon cancer cohort

After Xylol-based deparaffinization of the FFPE samples (2-4 10 µm FFPE tissue sections per sample), genomic DNA was extracted using a DNeasy Blood & Tissue Kit (Qiagen; Cat. No 69504/69506 (Qiagen GmbH, Vienna, Austria), following the manufacturer’s protocol. DNA purity was measured using a NanoDrop spectrophotometer (Thermo Fisher Scientific, Vienna, Austria). DNA concentrations were determined by Qubit fluorimetry (Thermo Fisher Scientific, Vienna, Austria).

Quantitative PCR was carried out using TB Green Premix Ex Taq II (TAKARA Bio, Japan) to determine the presence of Fn (NusG gene of Fn and PCBP1 gene as a human internal control). Primers were specific for Fn, and no cross-reactivity was observed for Fusobacterium necrophorum (**Supplementary Material, Table S1**). At least 10 ng of genomic DNA was used as the template. All reactions were conducted in duplicate in a LightCycler 96 thermocycler (Roche) for 50 cycles [initial denaturation (95°C, 30s), 50x denaturation (95°C, 5s), annealing (58°C, 10s) and extension (72°C, 30 s); final extension (72°C, 60s) and melting analyses (95°C for 10s, 65°C for 60s, 97°C for 1s)]. Product specificity was confirmed by gel electrophoresis, sequencing, and melting curves. At least 15% of patient samples were subjected to replication, yielding very similar results. Samples with poor DNA quality or unclear melting curves were excluded (n=25), leaving 60 samples for the colon cancer cohort.

To determine the PCR sensitivity for Fn, DNA from a negative patient was spiked with a range of 0.3 ng Fn gDNA (~128,000 DNA copies) to 3x10-70.0000003 ng (0.18 copies). At least 100 copies of bacterial DNA were reliably detected using PCR. Burden classification was then performed by calculating the ratio between bacterial and human cells.

#### 2.3.3 Rectal cancer cohort

Four 10 µm FFPE tissue sections per sample were deparaffinized with deparaffinization solution from Qiagen (cat. No. 19093). Total RNA was extracted using an RNeasy FFPE Kit (cat. No. 73504; Qiagen GmbH, Vienna, Austria), following the manufacturer’s recommendations, including DNAse-1 treatment. RNA purity was measured using a NanoDrop spectrophotometer (Thermo Fisher Scientific, Vienna, Austria). No residual genomic DNA was found after analyzing RNA purity using Qubit fluorimetry (Thermo Fisher Scientific, Vienna, Austria). RNA integrity was evaluated using Bioanalyzer pico-RNA technology (Agilent Technologies GmbH, Vienna, Austria). RNA was reverse-transcribed using the RevertAid H Minus First Strand cDNA Synthesis Kit (Thermo Fisher Scientific, USA) with random hexamers, according to the manufacturer’s instructions. 0.1 M NaOH and HCl were added for degradation of the template RNA and neutralization. A minimum of 300 ng RNA was used per sample.

qPCR was performed using the same cycle conditions as those for the colon cancer cohort, except for the use of an annealing temperature of 61°C. Between 40 and 125 ng of cDNA was used as a template for each reaction. Poor-quality samples were excluded from the cohort (n=14), resulting in n=47.

The quantification cycle (Cq) values for Fn NusG were normalized to the Cq values of the human housekeeping gene RPLp0 present in the biopsy. The fold-difference (2-(Cq Fn NusG – Cq RPLp0) was calculated by subtracting the Cq values of human DNA from the Cq values of Fn.

Details are described in Supplementary Methods (**Supplementary Material**, **Supplementary Figures S4**, **S5**).

#### 2.3.4 Bulk RNA-sequencing analysis

##### 2.3.4.1 Preprocessing

A total of 33 RNA sequencing samples from microsatellite-stable rectal adenocarcinomas were preprocessed and mapped to the human reference genome (hg38/GRCh38) using version 3.14.0 of the nf-core/rnaseq pipeline (19, 20). Briefly, reads were trimmed with Trimgalore and aligned to the reference genome using STAR (21) with GENCODE v46 annotations. Gene expression was quantified using the Salmon method (22). The resulting gene count tables were further processed using R version 4.4.1. For subsequent analyses, only samples with high (13 Fn-negative (Fn^−^) and 6 Fn-positive (Fn^+^)) and/or good (9 Fn^−^ and 4 Fn^+^) confidence of Fn measurements were included, yielding 22 Fn^−^ and 10 Fn^+^ samples.

##### 2.3.4.2 Differential gene expression and gene set enrichment analysis

Differential gene expression analysis between Fn^+^ and Fn^−^ samples was performed using DESeq2 v.1.44.0 (23), incorporating sex as a covariate in the linear model. Genes were defined as differentially expressed based on thresholds of |log_2_ fold change| > 1 and an adjusted p-value < 0.1. Volcano plots were generated using the EnhancedVolcano package v.1.22.0 to visualize differential expression results. Gene Set Enrichment Analysis was performed using clusterProfiler package v.4.12.6, leveraging the Gene Ontology-Biological Process database.

##### 2.3.4.3 Imaging mass cytometry and data analysis

IMC was performed on tissue microarrays from 19 microsatellite-stable CRC samples (10 Fn^−^, 9 Fn^+^) as previously described (24, 25), using an antibody panel (**Supplementary Material**, **Supplementary Table S2**). Per sample, one or two 1,000x1,000µm regions of interest (ROIs) were ablated depending on tissue availability. Images were visually inspected and exported to ome.tiff using MCD™ viewer software (standard biotools). Image normalization, cell segmentation, and phenotype identification were performed as described previously (24) and the steps were validated using the original images.

ROIs for IMC were selected within tissue microarray cores constructed in collaboration with a pathologist to ensure representative tumor regions, focusing on areas containing both vital tumor cells and stroma. ROIs were chosen based on high tumor cellularity and exclusion of necrotic, folded, or degraded tissue. Selection was performed blinded to Fn status to minimize selection bias. Per sample, one or two ROIs of approximately 1 mm^2^ were analyzed.

Phenotype classification was performed using unsupervised k-means clustering. Initially, data were over-clustered, and clusters were manually merged based on overlapping protein expression profiles. A minimal signal threshold of 0.1 (scaled between 0 and 1) was applied during merging. All resulting clusters were quality-checked against raw image files to confirm co-expression within single cells and exclude artifacts from overlapping cells. A heatmap of marker expression per phenotype was used to validate cluster identity and can be interpreted as a confusion matrix.

Only samples with clearly defined Fn status were included in the analysis: Fn^+^ samples with high or medium bacterial burden and Fn^−^ samples with no detectable burden. Samples with low burden were excluded due to ambiguity in classification.

##### 2.3.4.4 Analysis of spatial cell organization using imaging mass cytometry data

To analyze the spatial organization of the cells, we constructed Voronoi diagrams based on the center coordinates of all the detected nuclei. Each Voronoi region is assigned to the corresponding cell type. Direct cell-cell interactions were defined as those with a shared Voronoi edge and a nucleus center-to-center distance of less than 100µm. To assess immune cell infiltration, we calculated the immune cell-tumor cell mixing score for each sample. This score was determined by dividing the number of direct immune cell-tumor cell interactions by the total number of direct immune cell interactions. This approach has also been employed to quantify the interactions between antigen-presenting cells (dendritic cells, macrophages, B cells, plasma cells, and tumor cells) and T cells (CD8+ cytotoxic T cells and CD4+ helper T cells). The statistical significance of the differences between Fn^+^ and Fn^−^ was assessed using the Wilcoxon test.

## 3 Results

### 3.1 Patient characteristics

**Figure 1:**
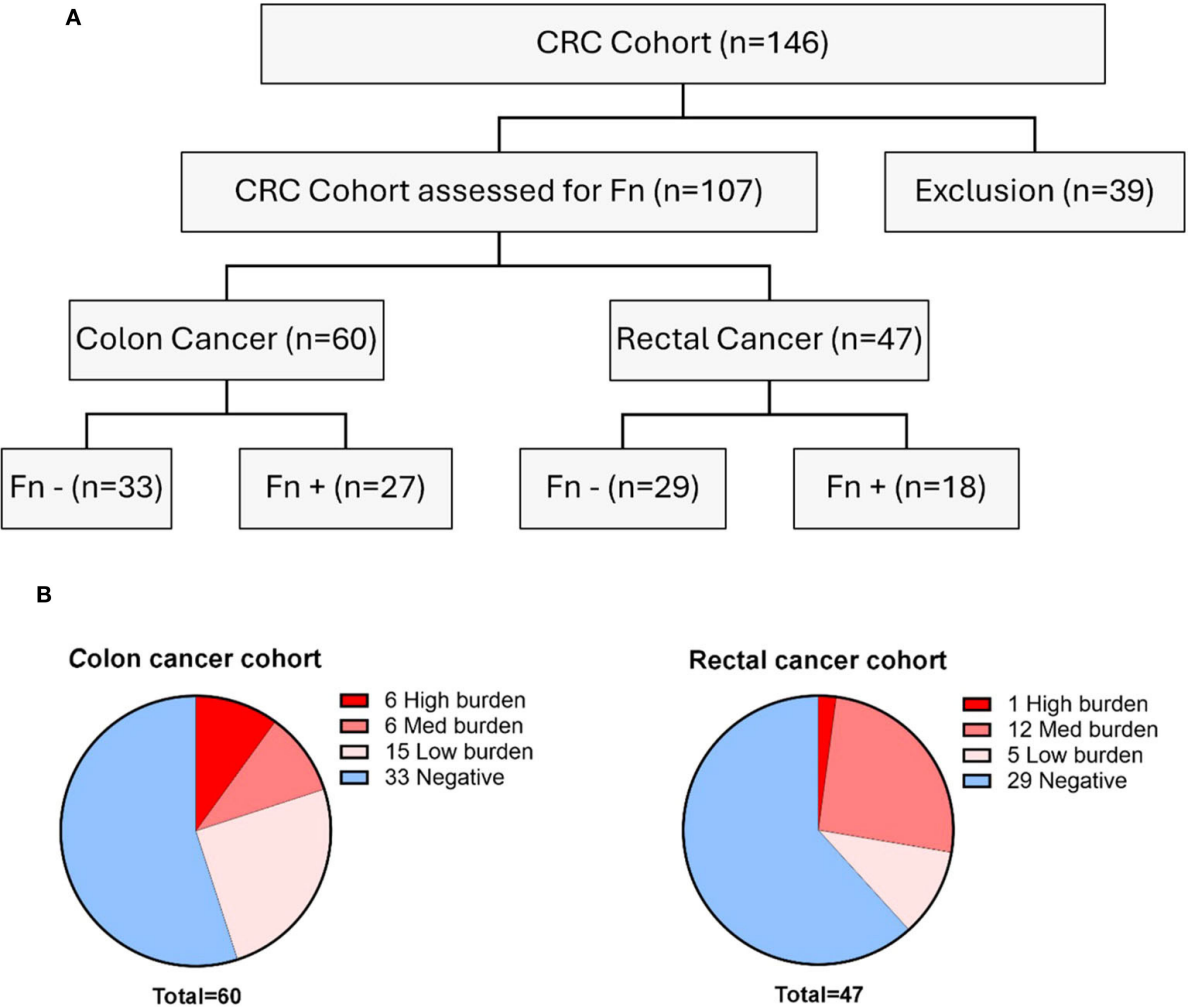
Distribution of patients in the cohort. **(A)** Overview of the two cohorts in terms of Fn status (gDNA) Samples with insufficient DNA quality or quantity were excluded. **(B)** Distribution of the bacterial burden in the two cohorts. CRC, colorectal cancer; Fn, *Fusobacterium nucleatum*; Cq, Quantification cycle.

In the entire cohort, 45 patients were classified as Fn^+^ and 62 as Fn- based on the qPCR results. The classification of bacterial burden is based on the ratio of human genome copies to Fn genome copies (ratio: 2^-(Cq Fn - Cq PCBP1)). Patients were classified according to high and low bacterial burdens in comparison with human DNA copies (**Figure 1**).

**Table 1** depicts the patient characteristics and tumor characteristics of the 107 CRC patients based on the bio tools Fn status determined by PCR. The average age at diagnosis was 62.5 years (range: 28–84 years), with a male preponderance (61%). Most patients (69%) had left-sided tumors (from the rectum to the distal third of the transverse colon). The tumors were predominantly classified as UICC stage III (89%). Histological grades 1 and 2 were the most frequently detected (65%). Among the 47 patients with rectal cancer, MSI/dMMR was detected in three patients (6%), whereas microsatellite/mismatch-repair status could not be analyzed in 12 (26%). Seven patients showed complete pathological response (pCR) following neoadjuvant chemoradiotherapy (15%). A statistically significant difference between Fn^+^ and Fn^−^ CRC was found with respect to tumor grade (p = 0.008), indicating that poorly differentiated CRC cases were more likely to be Fn^+^. These results align with previous findings, implying that Fn^+^ CRCs more often present with higher tumor grades (26, 27). The remaining baseline patient and tumor characteristics did not differ between the Fn^+^ and Fn^−^ patients.

**Table 1:** Baseline characteristics. Chi-squared tests for clinical, pathological, and molecular features between patients with Fn^+^ and Fn^−^ Rectal and Colon Cancer.

Variables	All cases, n (%)		Fn Status (n=107)				*p-value*
			*Fn pos, n (%), n=45*		*Fn neg, n (%), n=62*		
Patient characteristics								
Age at Diagnosis (years + range)	62.5	(28-84)	60.6	(28 -79)	63.9	(46-84)	0.336
Sex								
Female	42	(39)	20	(19)	22	(21)	0.349
Male	65	(61)	25	(23)	40	(37)	
Tumor characteristics								
UICC stage								
II	18	(17)	6	(6)	12	(11)	0.575
III	89	(83)	39	(37)	50	(47)	
Tumor location								
Right-sided	33	(31)	14	(13)	19	(18)	0.959
Left-sided	74	(69)	31	(29)	43	(40)	
Tumor differentiation								
G1-2	70	(65)	23	(22)	47	(44)	0.008
G3	37	(35)	22	(21)	15	(14)	
Metachronous metastases								
No	80	(75)	35	(33)	45	(42)	
Yes	27	(25)	10	(9)	17	(16)	0.541
Metastases distribution^#^								
Liver	17	(16)	7	(18)	10	(25)	0.936
Lung	8	(8)	3	(8)	5	(13)	0.786
Peritoneum	6	(6)	4	(10)	2	(5)	0.209
Distant Lnn	6	(6)	2	(5)	4	(10)	0.656
CNS	1	(1)	0	(0)	1	(3)	0.392
Bone	2		0	(0)	2	(5)	0.224
Molecular Features								
Microsatellite status*								
MSI/dMMR	5	(5)	3	(6)	2	(4)	0.225
MSS/pMMR	44	(41)	15	(31)	29	(59)	
Missing	58	(54)					
pCR^+^								
Yes	7	(15)	2	(4)	5	(11)	0.556
No	40	(85)	16	(34)	24	(51)	

#Multiple designations possible, + Only in Rectal Cancer Cohort. *Microsatellite status was assessed primarily in the rectal cancer cohort. MSI/dMMR, microsatellite instability-high/mismatch repair deficient; MSS/pMMR, microsatellite stability/mismatch repair proficient; Lnn, lymph nodes; CNS, central nervous system; pCR, pathological complete remission; Fn, *Fusobacterium nucleatum*.

### 3.2 Clinical outcome

The OS and DFS at ten years in the entire cohort (colon and rectal cancer) were 68% and 62%, respectively, as shown in **Supplementary Material**, **Supplementary Figure S1A** and **S1B**. Twenty-seven patients (25%) developed metachronous metastases during the follow-up period. The metastatic sites in descending order were the liver (16%), lungs (8%), distant lymph nodes (6%), peritoneum (6%), bones (2%), and central nervous system (1%). Local recurrence occurred in eight patients (8%), six of whom also developed distant metastases.

In the colon cancer subgroup, the ten-year OS and DFS at ten years were 68% and 61%, respectively (**Supplementary Material**, **Supplementary Figures S2A, B**), while in the rectal cancer subgroup, the corresponding rates were 65% and 63%, respectively (**Supplementary Material**, **Supplementary Figures S3A, B**).

**Figure 2:**
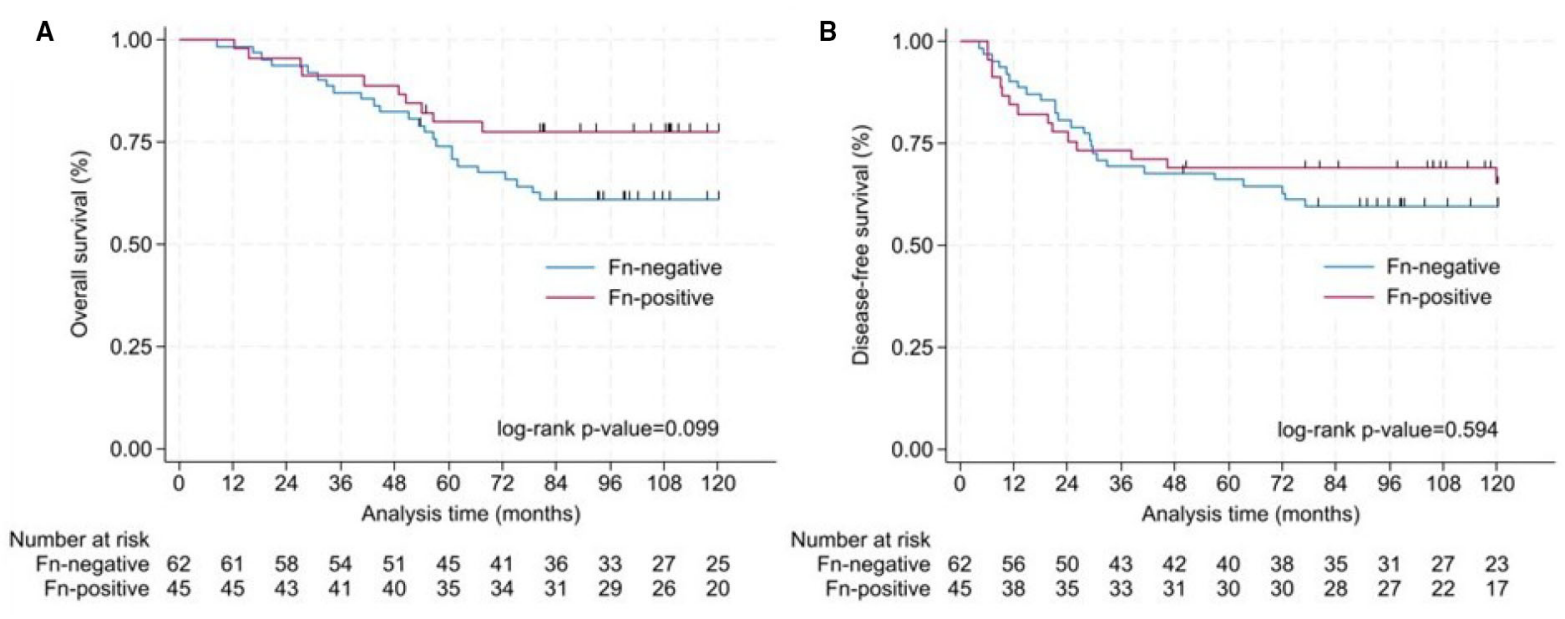
Analysis of OS and DFS in colorectal cancer patients. Survival differences between Fn^+^ and Fn^−^ were assessed using log-rank tests over the full follow-up period. **(A)** Kaplan–Meier curve for OS according to the detection of Fn in colorectal tissue. Ten-year OS was 78% for Fn^+^ and 61% for Fn^−^ (log-rank p = 0.099). **(B)** Kaplan–Meier curve for disease-free survival according to the detection of Fn in colorectal tissue. Ten-year disease-free survival was 65% for Fn^+^ and 60% for Fn^−^ (log-rank p = 0.594). Fn, *Fusobacterium nucleatum*; OS, overall survival; DFS, disease-free survival.

When stratified by Fn status, the presence of Fn in CRC cells did not significantly affect survival outcomes. The Kaplan–Meier curve in **Figure 2A** only showed a trend towards longer OS (log-rank p = 0.0990) in Fn^+^ patients (78%) compared to Fn^−^ patients (61%). Ten year DFS did not differ between groups, with 65% in Fn^+^ and 60% in Fn^−^ patients, as illustrated in **Figure 2B** (log-rank p = 0.5947). The log-rank test was performed across the entire follow-up period, comparing the full Kaplan–Meier survival curves between Fn^+^ and Fn^−^.

### 3.3 Histological findings

A subset of nine patients with Fn^+^ primary tumors as assessed by PCR were further analyzed by immunostaining. Within this illustrative cohort, five showed Fn^+^ primary tumor as well as regional lymph nodes by immunohistochemistry, three samples were not measurable due to insufficient FFPE quality, and one sample was negative for Fn by immunohistochemical despite PCR positivity.

#### 3.3.1 Fn persistence in metastases

**Figure 3:**
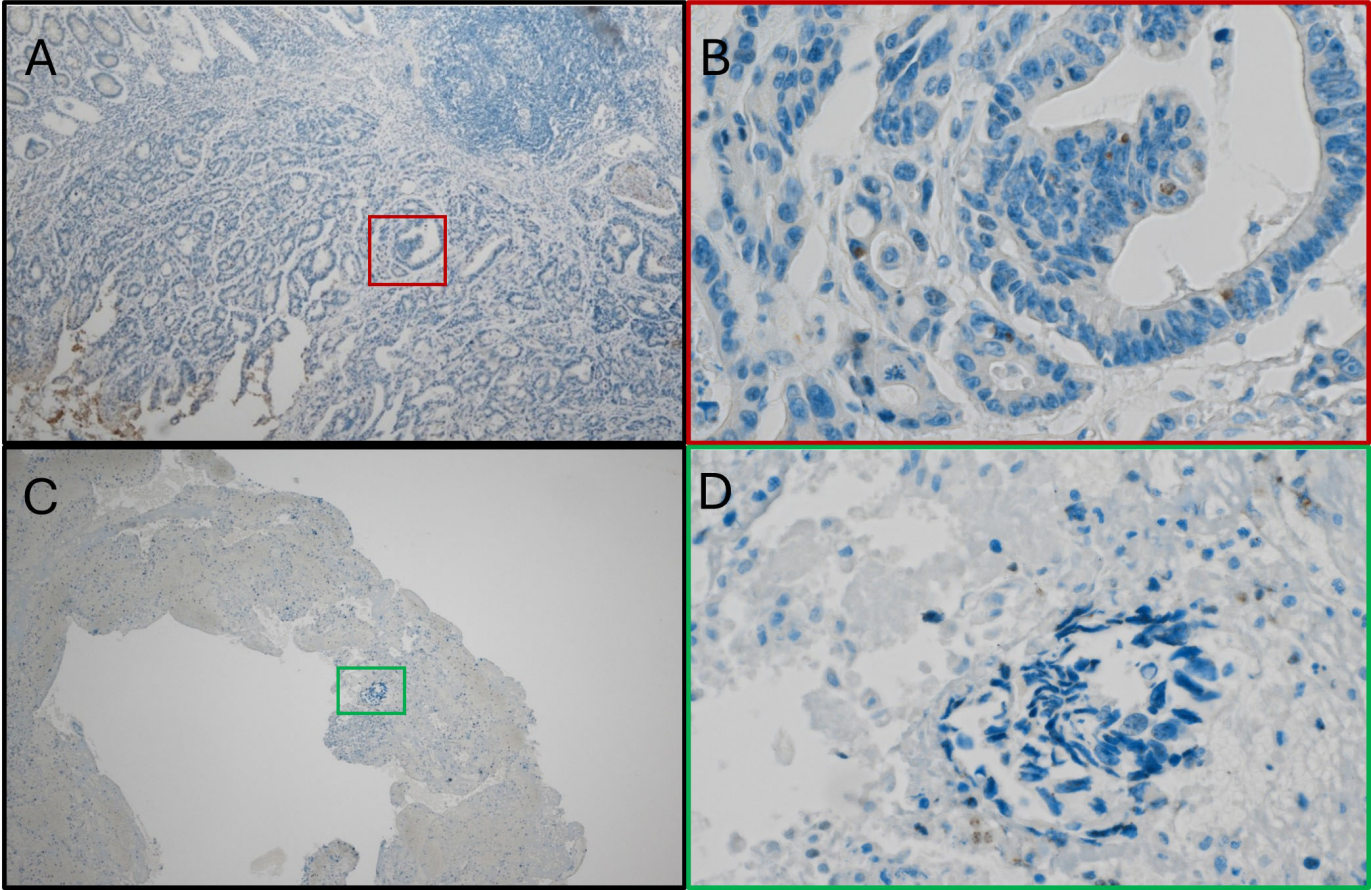
Fn persistence during cancer progression. **(A)** Immunostain with anti-Fn; 40x magnification; location: ascending colon; positive nuclear staining with the tumor cells. **(B)** Immunostainwith anti-Fn; 400x magnification; location: primary tumor; positive nuclear staining with the tumor cell. **(C)** Immunostain with anti-Fn; 40x magnification; location: liver; positive nuclear staining with the tumor cells. **(D)** Immunostain with anti-Fn; 400x magnification; location: liver; positive nuclear staining with the tumor cells. Fn, *Fusobacterium nucleatum*.

Among the nine patients with Fn^+^ primary tumors as assessed by PCR and IHC, three developed metachronous metastases. In all three cases, Fn was detected in both the primary tumor and metastatic sites, including distant lymph nodes, peritoneum, and liver (**Figure 3**). Panels A and B show Fn-positive nuclear staining in the primary tumor, while Panels C and D demonstrate similar staining patterns in liver metastases.

#### 3.3.2 Extracellular location

**Figure 4:**
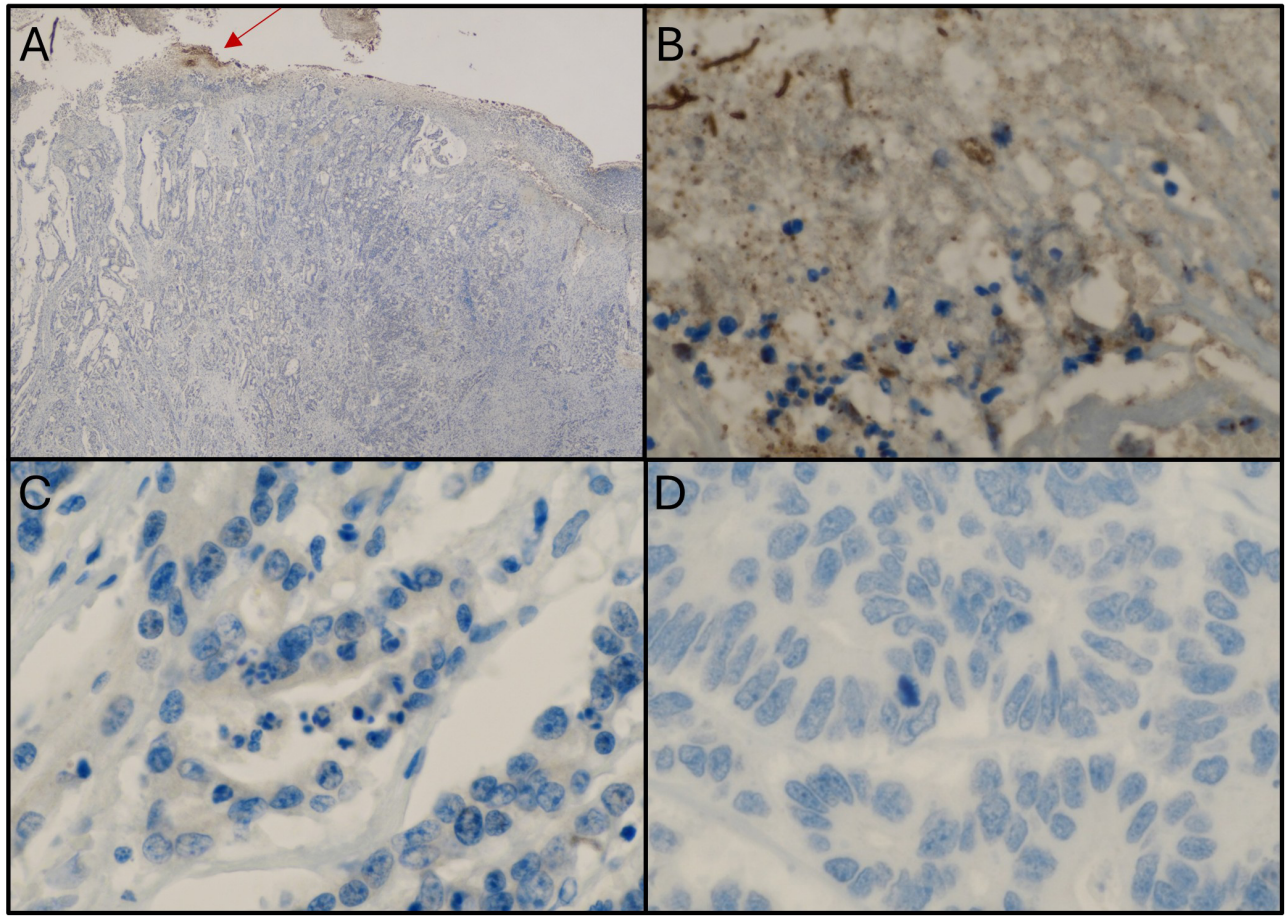
Samples from a patient with colorectal cancer showing an extracellular location of Fn. **(A)** Immunostaining with anti-Fn; 20x magnification; location: ascending colon; positive staining within the biofilm (red arrow). **(B)** Immunostaining with anti-Fn; 600x magnification (from picture A); location: primary tumor; positive staining within the biofilm. **(C)** Immunostaining with anti-Fn; location: primary tumor; 600x magnification; no positive staining in tumor cells. **(D)** Immunostaining with anti-Fn 600x magnification; location: liver metastasis; no positive signal in tumor cells. Fn, *Fusobacterium nucleatum*.

**Figure 4** illustrates a representative case of Fn^+^ CRC with extracellular localization of the bacterium. Panel A shows positive immunostaining for Fn on the ulcerated luminal surface of the tumor (20x magnification), while Panel B (600x) confirms Fn presence within the biofilm. Notably, Panels C and D show no Fn signal in the tumor cells of the primary tumor or liver metastasis, despite a high bacterial load detected by qPCR.

#### 3.3.3 Mesenchymal phenotype

**Figure 5:**
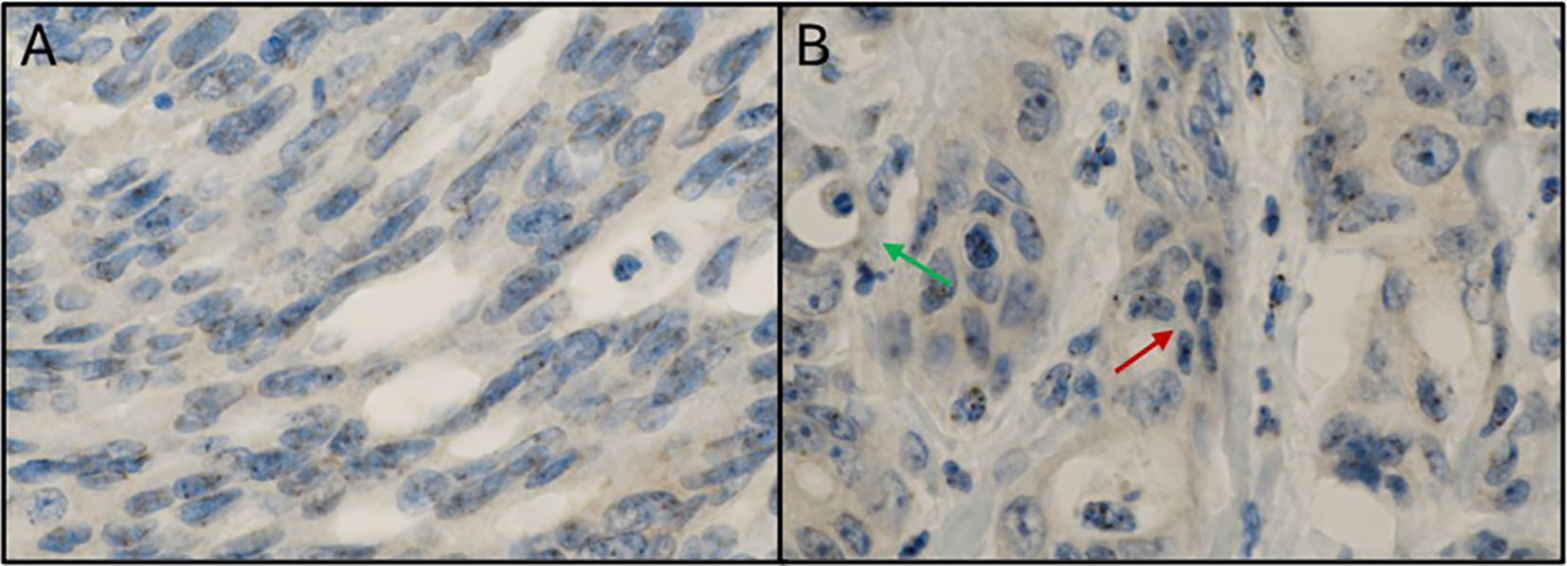
Mesenchymal phenotype in Fn positive colorectal cancer. **(A)** Immunostaining with anti-Fn; 600x magnification; location: ascending colon; tumor cells with a mesenchymal phenotype (elongated cell bodies and nuclei) with positive signals within the nuclei. **(B)** Immunostaining with anti-Fn; 600x magnification; location: peritoneal metastasis; tumor cells with positive signals within the nuclei. Some tumor cells show a mesenchymal phenotype (elongated cell bodies and nuclei; red arrow) between glandular tumor cell formations with an „ordinary” cell phenotype (green arrow). Fn, *Fusobacterium nucleatum*.

**Figure 5** presents Fn^+^ CRC cells exhibiting cytomorphological features consistent with epithelial-to-mesenchymal transition (EMT). In Panel A, tumor cells from the ascending colon show elongated nuclei and positive nuclear Fn staining. Panel B shows peritoneal metastasis with both mesenchymal-like and glandular tumor cell phenotypes, with Fn signals localized to the nuclei of mesenchymal-like cells.

### 3.4 Molecular genetic findings

#### 3.4.1 Mixing score

**Figure 6:**
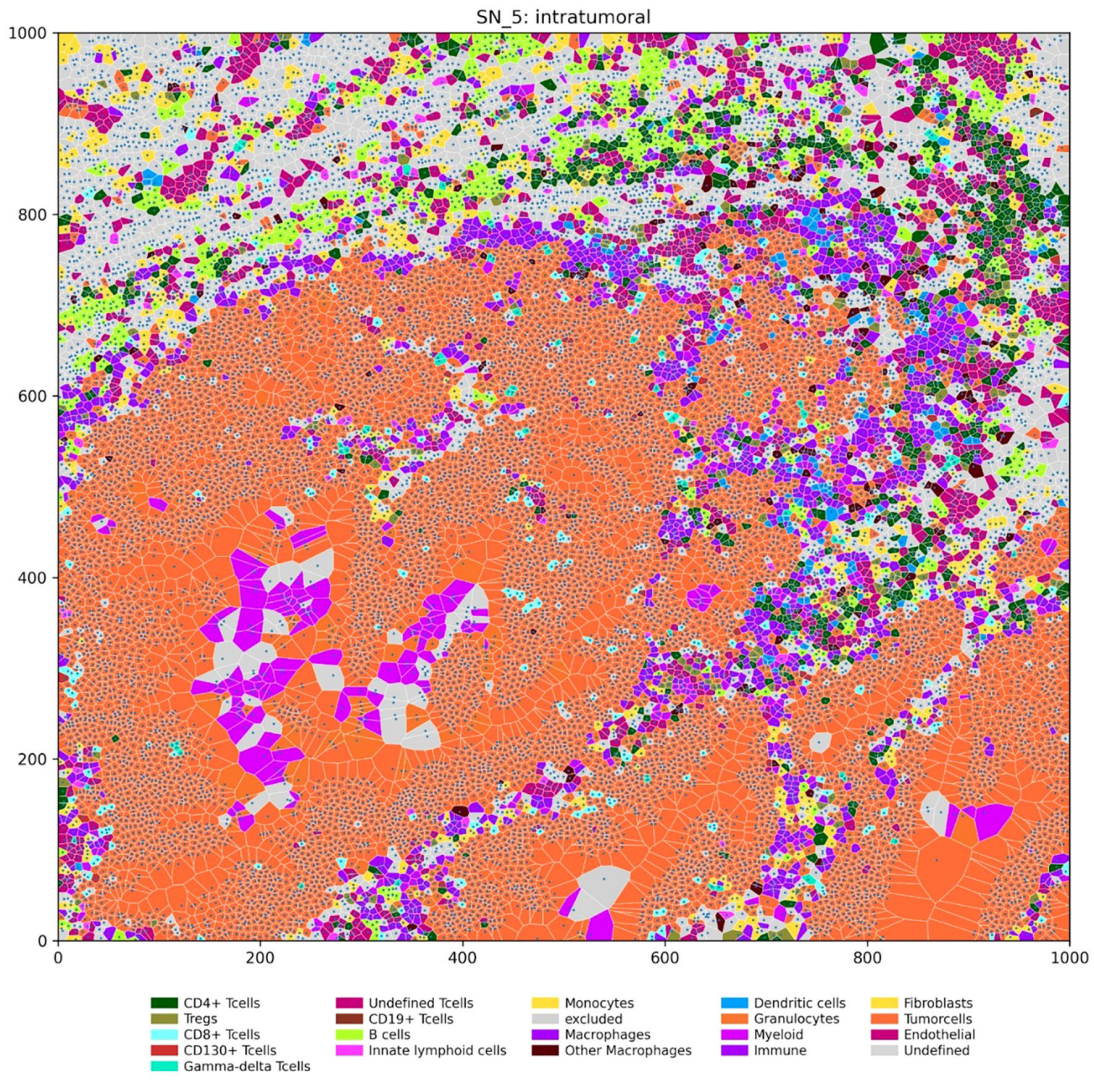
Spatial distribution of immune and tumor cells in an intratumoral region (units: µm). The Voronoi diagram depicts the spatial organization of various cell types within a representative intratumoral region of a tumor sample. Each polygon represents a single cell, with its location determined by the nucleus center coordinates. Cells are color-coded according to their type, as indicated in the legend.

To investigate the spatial relationships between different cell types within the tumor microenvironment, we performed IMC on tissue microarrays from Fn^+^ and Fn^−^rectal cancer MSS/pMMR samples. Regions analyzed included intratumoral (Fn^+^ n=7, Fn^−^ n=6) and invasive margins (Fn^+^ n=7, Fn^−^ n=9). We then employed Voronoi tessellation, a geometric method used to define cell boundaries based on nuclei positions, allowing quantification of direct cell–cell interactions by assessing the shared edges of the Voronoi polygons. **Figure 6** provides a visual example of this spatial mapping of immune and tumor cells.

To quantify specific cell-cell interactions within the tumor microenvironment, we adapted a method from Keren et al. (28) and developed two mixing scores, which represent the proportion of immune cells interacting directly with tumor or antigen-presenting cells. These scores quantify the degree of interaction between different cell types (**Supplementary Material**, **Supplementary Table S3**) based on their proximity to the tissue.

**Figure 7:**
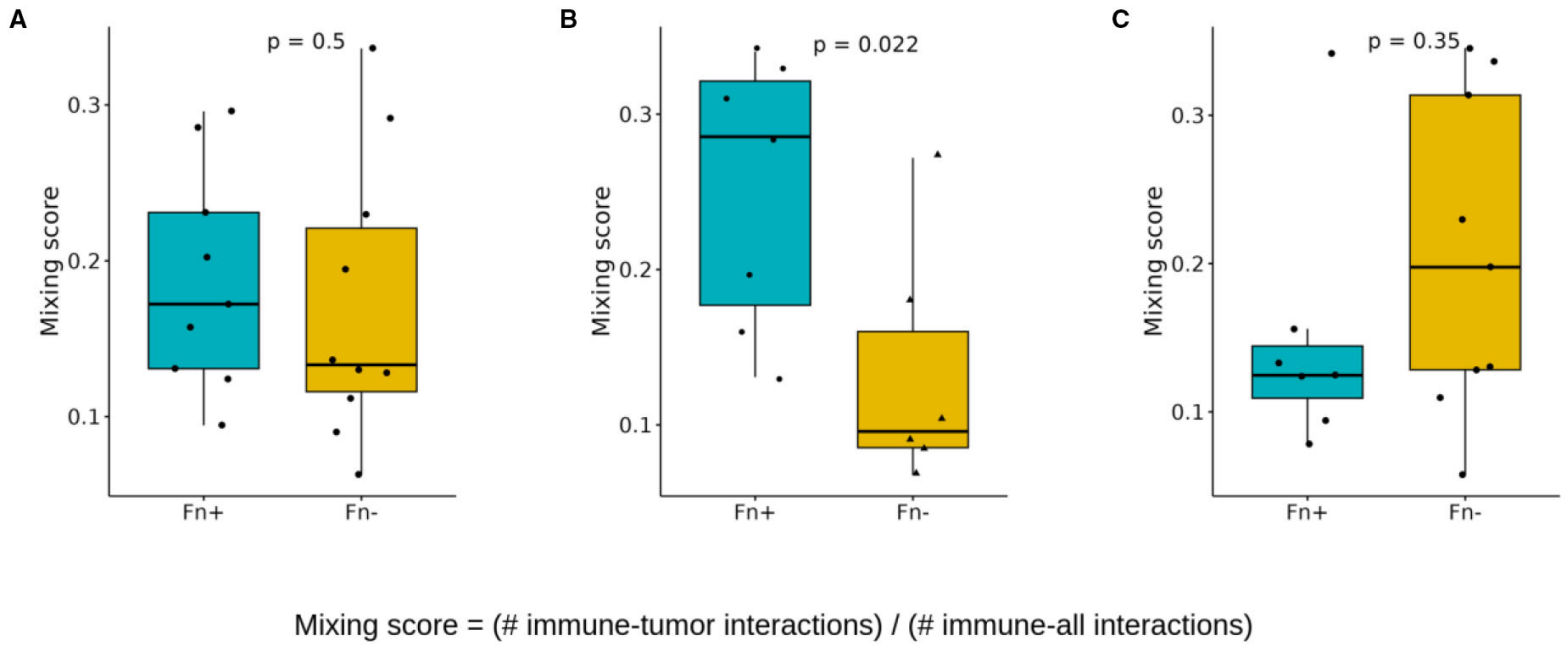
Spatial cell-cell interactions: mixing scores. Boxplots showing differences in the immune/tumor mixing scores (immune/tumor interactions versus immune any cell interaction) between Fn^+^ and Fn^−^ patients **(A)** location independent **(B)** intratumoral **(C)** invasive margin. These mixing scores quantify the immune cell infiltration in each patient sample and are determined by dividing the number of direct immune cell-tumor cell interactions by the total number of direct immune cell interactions (p-values calculated with Wilcoxon test). Fn^+^, *Fusobacterium nucleatum* positive; Fn^−^, *Fusobacterium nucleatum* negative.

The immune cell-tumor cell mixing score was calculated to assess the extent of immune cell infiltration and their interaction with tumor cells, as shown in **Figure 7**. This score represents the ratio of direct immune cell-tumor cell contacts to the total number of immune cell interactions. Statistical analysis revealed that while overall immune cell–tumor cell mixing across all tissue regions showed no significant difference in Fn^+^ tumors (**Figure 7A**), a significant increase was observed specifically in intratumoral regions. Fn^+^ tumors exhibited a higher degree of immune cell-tumor cell interactions within the tumor core, as illustrated in **Figure 7B**, suggesting increased immune infiltration and potential engagement with tumor cells. No significant differences were observed at the tumor margins (**Figure 7C**), indicating similar levels of interaction at the periphery in both the Fn^+^ and Fn^−^ groups.

**Figure 8:**
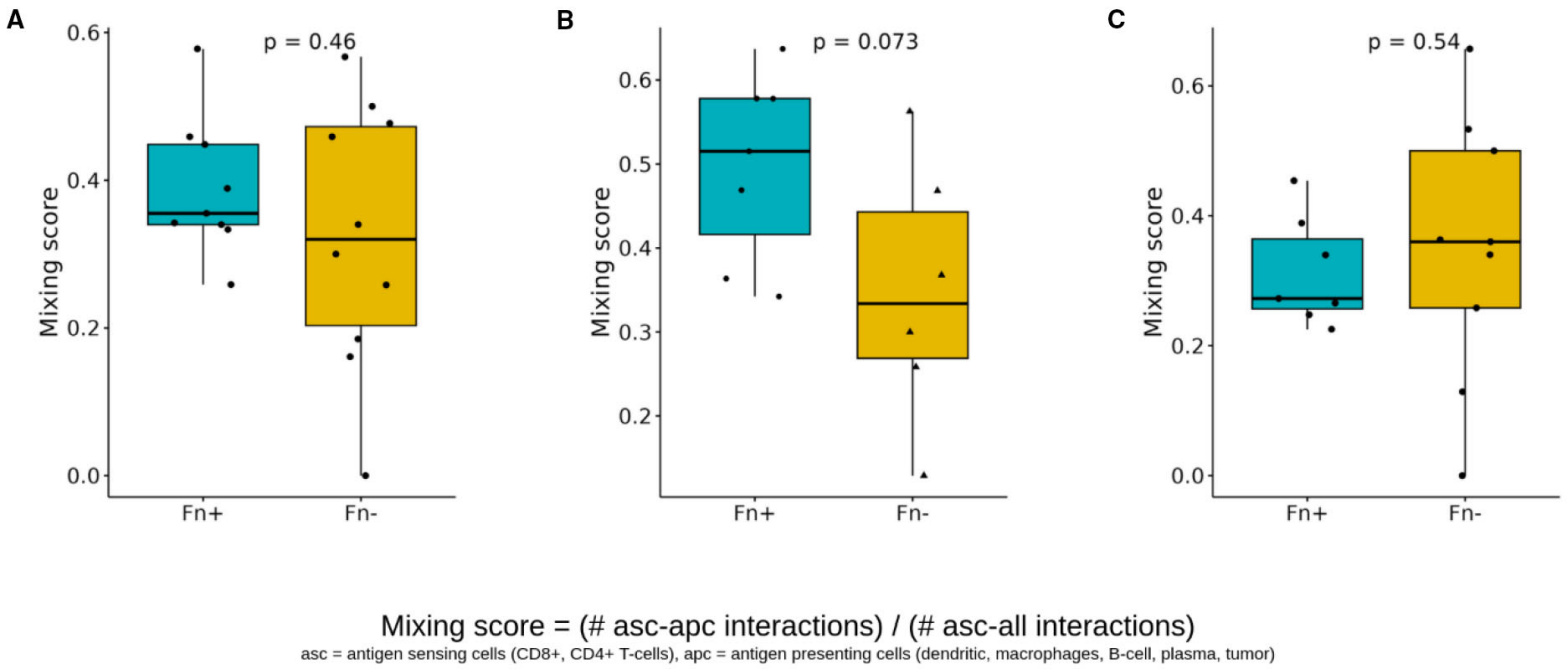
Spatial cell-cell interactions of antigen sensing and antigen-presenting cells. Boxplots showing differences in the antigen sensing/presenting mixing scores (CD8+/CD4+ T cells with APC interactions versus CD8+/CD4+ with cell interaction) between Fn^+^ and Fn^−^ patients **(A)** location-independent **(B)** intratumoral **(C)** invasive margin. These mixing scores quantify the spatial interaction of antigen-presenting cells (dendritic cells, macrophages, B cells, plasma cells, and tumor cells) and antigen-sensing cells (CD8+ T cells, CD4+ T cells), and are determined by dividing the number of direct interactions of apc with asc by the total number of direct cell-cell interactions of asc (p-values calculated with Wilcoxon test). Fn^+^, Fusobacterium nucleatum positive; Fn^−^, Fusobacterium nucleatum negative; asc, antigen sensing cells; apc, antigen presenting cells.

Second, we quantified the interactions between antigen-presenting cells (APCs) and T cells (CD8+ and CD4+) using a similar scoring approach (**Figure 8**). The APC-T cell mixing score showed no significant increase in Fn^+^ tumors overall (**Figure 8A**) but showed an evident elevation within the intratumoral regions (**Figure 8B**). Again, no significant differences were observed at tumor margins (**Figure 8C**).

#### 3.4.2 High prevalence of Fn is linked to suppression of immune-related pathways

To examine the transcriptional differences in CRC with either Fn^+^ or Fn^−^ MSS/pMMR samples, we performed differential gene expression analyses from the bulk RNA-sequencing data, as mentioned above, and identified 85 genes with statistically significant different expression levels. (**Supplementary Material**, **Supplementary Figure S6**).

**Figure 9:**
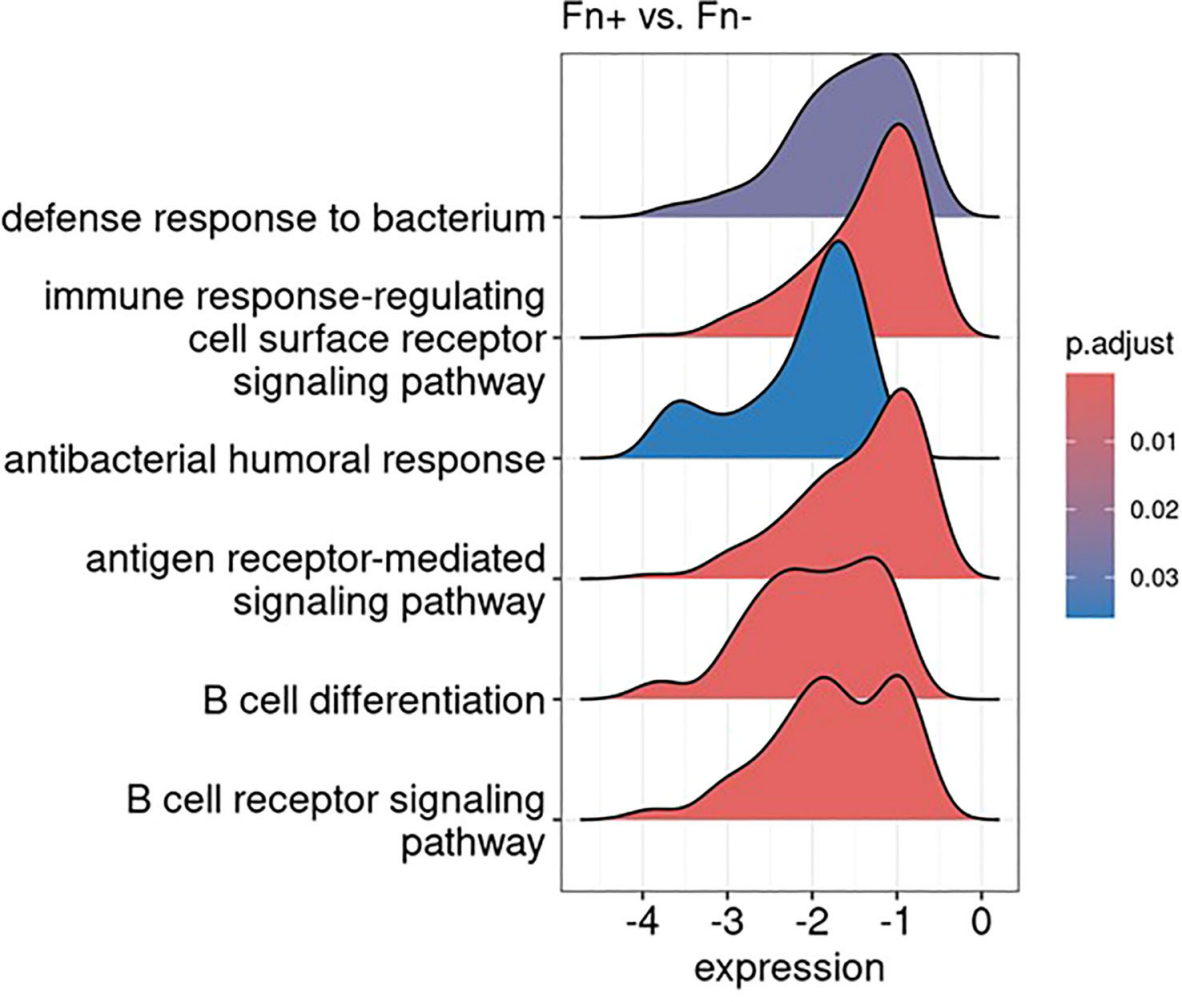
Gene set enrichment analysis reveals downregulation of immune-related pathways: results visualized as ridge plots, illustrating the distribution of selected significantly suppressed gene ontology biological processes terms (adjusted p-value < 0.1) in Fn^+^ versus Fn^−^ samples. The x-axis represents the log2 fold change in gene expression of the genes involved in these terms, while the y-axis indicates the density. These terms were chosen based on their biological relevance and significance in the context of the analysis. Fn^+^, *Fusobacterium nucleatum* positive; Fn^−^, *Fusobacterium nucleatum* negative.

To further explore the functional impact of these transcriptional changes, we conducted Gene Set Enrichment Analysis using the Gene Ontology-Biological Process database. Immune-related pathways were prominently suppressed in Fn^+^ samples, with significant downregulation of the “immune response-regulating cell surface receptor signaling pathway”, “antigen receptor-mediated signaling pathway,” and “B cell receptor signaling pathway”, as visualized in **Figure 9** using ridge plots.

Additionally, Gene Set Enrichment Analysis results indicated a pronounced downregulation of pathways involved in antibacterial defense mechanisms, which is consistent with the intracellular bacterial context observed in Fn^+^ samples. **Figure 9** summarizes the transcriptional suppression of immune and antibacterial pathways in Fn^+^ tumors.

## 4 Discussion

The clinical survival data of this cohort (**Figure 1**), comprising mainly stage III CRC patients (n=89) (**Table 1**), corroborate the findings of Salvucci et al. in their analysis of an unselected Fn group (14) and align with previous studies (29–31). Previous studies suggest that biofilms enhance bacterial persistence and resistance, contributing to an inflammatory tumor microenvironment (32). Fn, a facultative intracellular bacterium, can survive both inside and outside host cells (33), though most molecular analyses do not distinguish between these localizations (8, 16, 34–36). This finding may be of interest for further studies, as it suggests potential variability in the intra- and extracellular effects of Fn in CRC patients.

Fn modulates the tumor microenvironment by suppressing anti-tumor immunity and promoting pro-inflammatory responses. This includes an increase of M2 macrophages, myeloid-derived suppressor cells, and Th17 cells, alongside a reduction of CD4+ and CD8+ T cells (7, 37–39). Fn abundance has been linked to PD-L1 upregulation as well as poor response to checkpoint inhibition (6). Intracellularly, Fn may induce chemoresistance via exosome-mediated drug efflux (40) and activation of oncogenic pathways such as Wnt/β-catenin, promoting EMT and metastasis (41–43).

Although spatial analysis was limited by sample size, Fn^+^ tumors demonstrated increased immune–tumor–APC interactions within the tumor core, suggesting potential, but possibly dysfunctional, T cell activation (**Figure 8**). This was accompanied by suppression of antibacterial defense pathways in gene expression analysis, which may support Fn persistence by reducing host antimicrobial responses (**Figure 9**). Notably, these immunological alterations did not translate into significant differences in OS or DFS, highlighting a potential disconnect between immune cell proximity and effective antitumor immunity.

To reconcile this apparent contradiction with the observed downregulation of immune-related pathways, we propose a model of spatially compartmentalized immune dysfunction: Fn^+^ tumors may exhibit T-cell exclusion at the invasive margin, limiting immune cell access, while functional impairment of immune cells within the tumor core prevents effective antitumor responses. This dual mechanism could explain the increased proximity of immune cells without corresponding activation, and warrants future functional validation in prospective studies.

Our observation of Fn persistence in colorectal metastases (**Figure 3**) supports the findings by Casasanta et al., who also demonstrated the potential of Fn to metastasize within cancer cells (18). Greco et al. further suggest that Fn is associated with MSI/dMMR and CIMP phenotype tumors and contributes to immune evasion and chemoresistance (44). These findings, together with our own, support a model in which Fn not only persists in metastatic lesions but also contributes to a tumor microenvironment that favors immune escape and therapeutic resistance.

While broad-spectrum antibiotics can eliminate Fn, concerns about resistance and microbiota disruption highlight the need for selective approaches (45–48). Promising preclinical strategies include bacteriophage ØTCUFN3 and antimicrobial FP-100, which have shown potential to target Fn without harming microbial diversity in experimental models (49, 50). Although these approaches are promising in preclinical models, they remain investigational and have not yet been evaluated in clinical trials. Future studies will be essential to evaluate their safety, efficacy, and therapeutic relevance in CRC patients.

## 5 Limitations

Despite these interesting findings, several limitations of this study should be considered when interpreting the results. First, the study’s relatively small sample size limits its statistical power and may impact the generalizability of the findings. Additionally, patient heterogeneity regarding tumor location (colon vs. rectum), stage, and treatment (e.g. adjuvant chemotherapy versus neoadjuvant chemoradiotherapy) may introduce variability that could affect the outcomes observed.

The detection of intracellular Fn in CRC tissues by immunostaining can be challenging because of the low bacterial load within the tumor tissues, potentially leading to false-negative results. Nuclear anti-Fn staining may further be non-specific and should be interpreted with caution. The use of different molecular templates and reference genes for Fn detection in colon and rectal cancer cohorts may introduce variability in bacterial burden quantification. Specifically, these approaches used distinct reference genes (PCBP1 for DNA-based detection in colon cancer and RPLP0 for RNA-based detection in rectal cancer), which may affect direct comparability between cohorts. While both assays were internally validated, direct comparisons between cohorts should be interpreted with caution. Future studies may benefit from harmonized detection protocols or cross-platform calibration.

Microsatellite/MMR status and BRAF/KRAS mutation data were unavailable for a substantial number of samples, primarily because routine testing of microsatellite/MMR status was not consistently implemented before 2017 and BRAF/KRAS mutation data are not routinely assessed in localized disease.

Furthermore, the retrospective nature of the study limits the ability to establish causal relationship, and findings should be considered exploratory and hypothesis-generating. Notably, the absence of significant survival differences despite pronounced immunological alterations may be attributed to confounding clinical variables such as treatment heterogeneity, tumor localization, and sample size limitations.

## 6 Conclusion and outlook

In summary, Fn contributes to an immunosuppressive microenvironment that impairs both antibacterial defense and antitumor immunity. Although the Fn+ status did not impact clinical outcome in this cohort, its role in promoting tumor proliferation, chemoresistance, and metastases development underscores the need for testing Fn-guided therapeutic strategies. Selective elimination of Fn may enhance the efficacy of existing therapies and reduce recurrence risk, but this hypothesis requires clinical validation through prospective studies.

## Data Availability

The datasets presented in this study can be found in online repositories. The names of the repository/repositories and accession number(s) can be found below: https://zenodo.org/records/15389290, 10.5281/zenodo.15389290.
