# Supplementary material for: Impact of Fusobacterium nucleatum on immune cell interactions and gene expression in colorectal cancer: a retrospective cohort study
Source: Front Immunol. 2025 Sep 11;16:1629014. doi: 10.3389/fimmu.2025.1629014 (PMC12460317; doi:10.3389/fimmu.2025.1629014)
Supplement: Supplementary file 1 [file DataSheet1.docx]

Supplementary Material

# Supplementary Figures and Tables

## Supplementary Figures


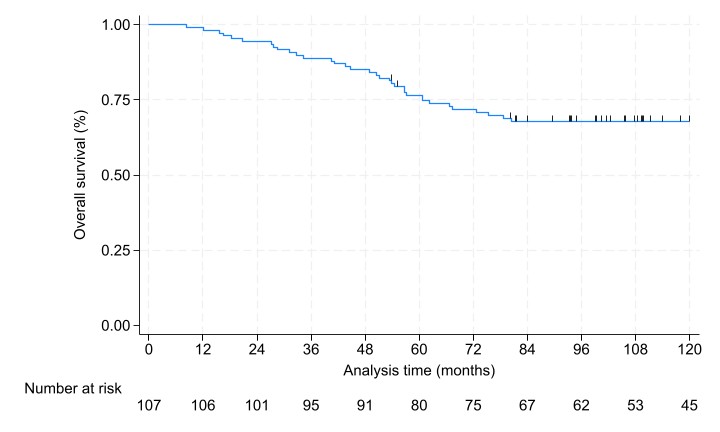
Supplementary Figure S1A. Kaplan–Meier curve for overall survival for the total cohort.

Ten-year overall survival was 68%.


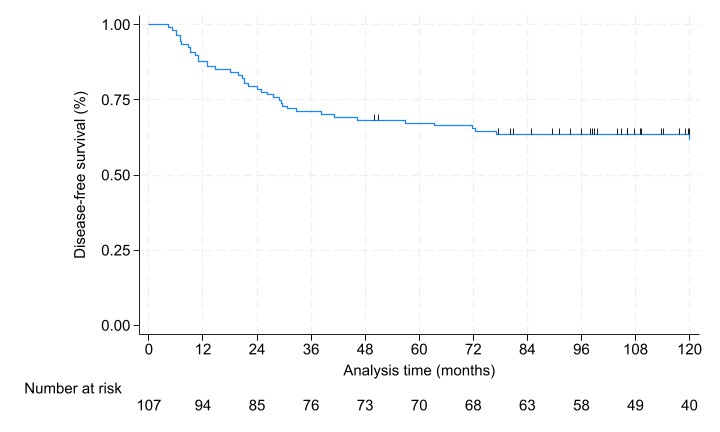


Supplementary Figure S1B. Kaplan–Meier curve for disease-free survival for the total cohort. Ten-year disease-free survival was 62%.


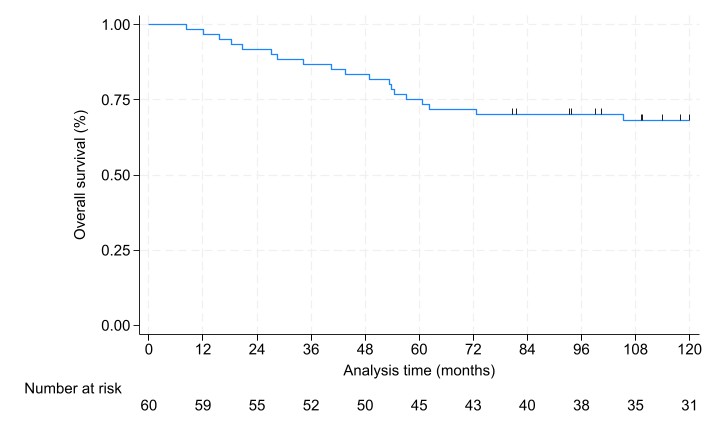


Supplementary Figure S2A. Kaplan–Meier curve for overall survival for the colon cancer cohort. Ten-year overall survival was 68%


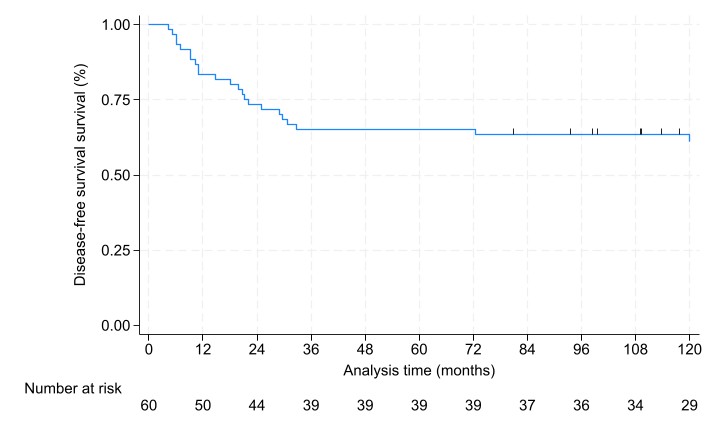
Supplementary Figure S2B. Kaplan–Meier curve for disease-free survival for the colon cancer cohort. Ten-year disease-free survival was 61%.


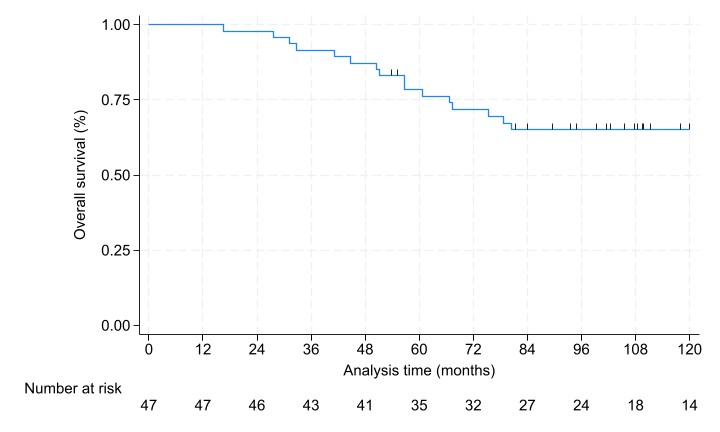
Supplementary Figure S3A. Kaplan–Meier curve for overall survival for the rectal cancer cohort. Ten-year overall survival was 65%.


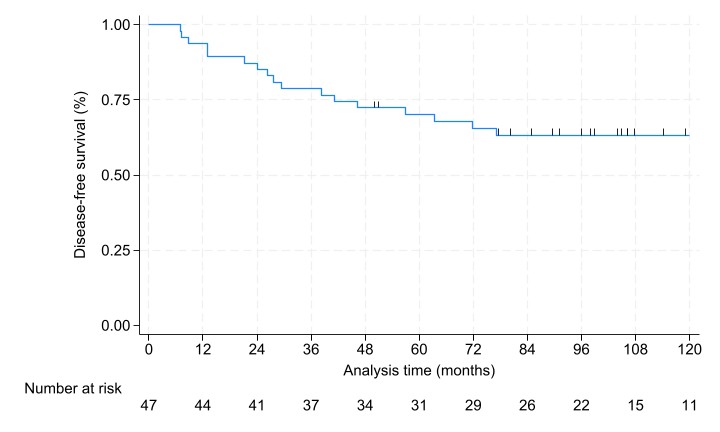
Supplementary Figure S3B. Kaplan–Meier curve for disease-free survival for the rectal cancer cohort. Ten-year disease-free survival was 63,3%.
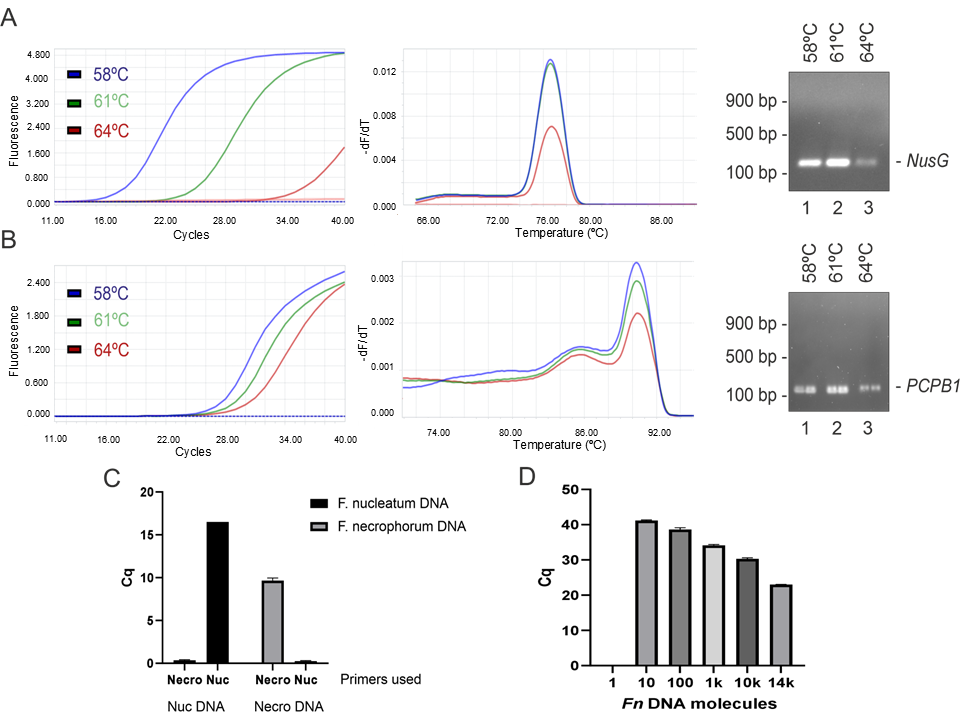
Supplementary Figure S4. Reaction optimization and limit of detection. A and B) qPCR analysis of NusG gene of Fn PCBP1 gene as human internal control showing Fn serial dilution and agarose gel of patient samples, each for both cohorts. C) Differentiation between Fn DNA and Fusobacterium necrophorum DNA. D) Sensitivity assay of Fn DNA molecules. Fn = Fusobacterium nucleatum.
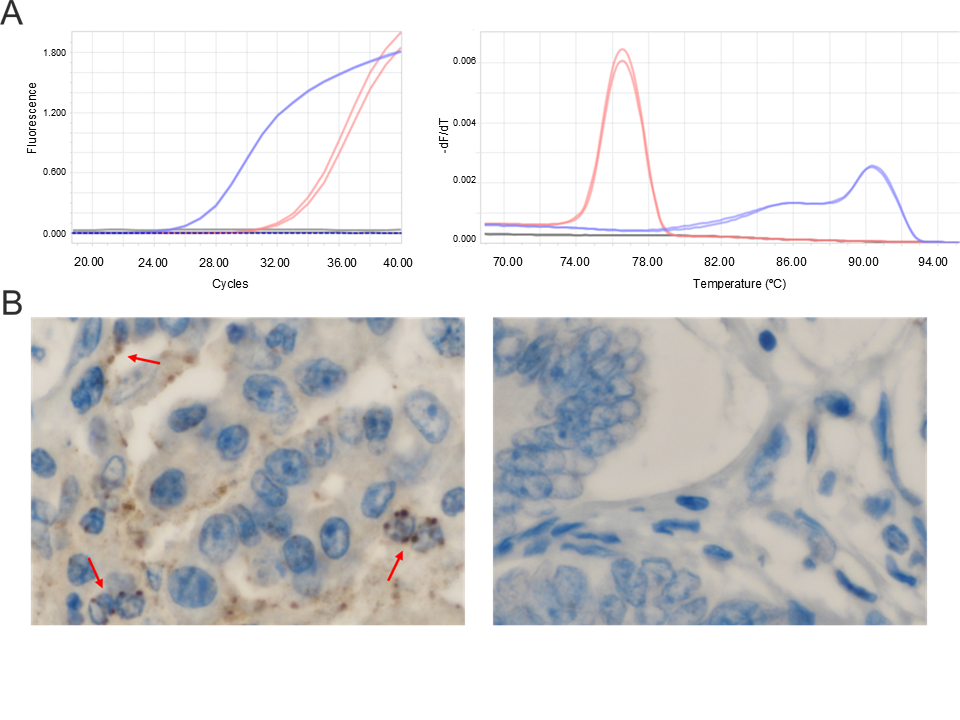
Supplementary Figure S5. Burden quantification. A) Exemplary amplification curves (left) and melting peaks (right) of human PCPB1 gene (blue) and Fn NusG gene (red). B) Representative immunohistochemical staining of FFPE sections. Left: primary tumor. Brown dots correspond to Fn colonizing the tissue (red arrows). Antibody: rabbit α Fn (Diatheva). Right: uninfected patient (negative on Fn. Fn = Fusobacterium nucleatum, FFPE = formalin-fixed paraffin-embedded


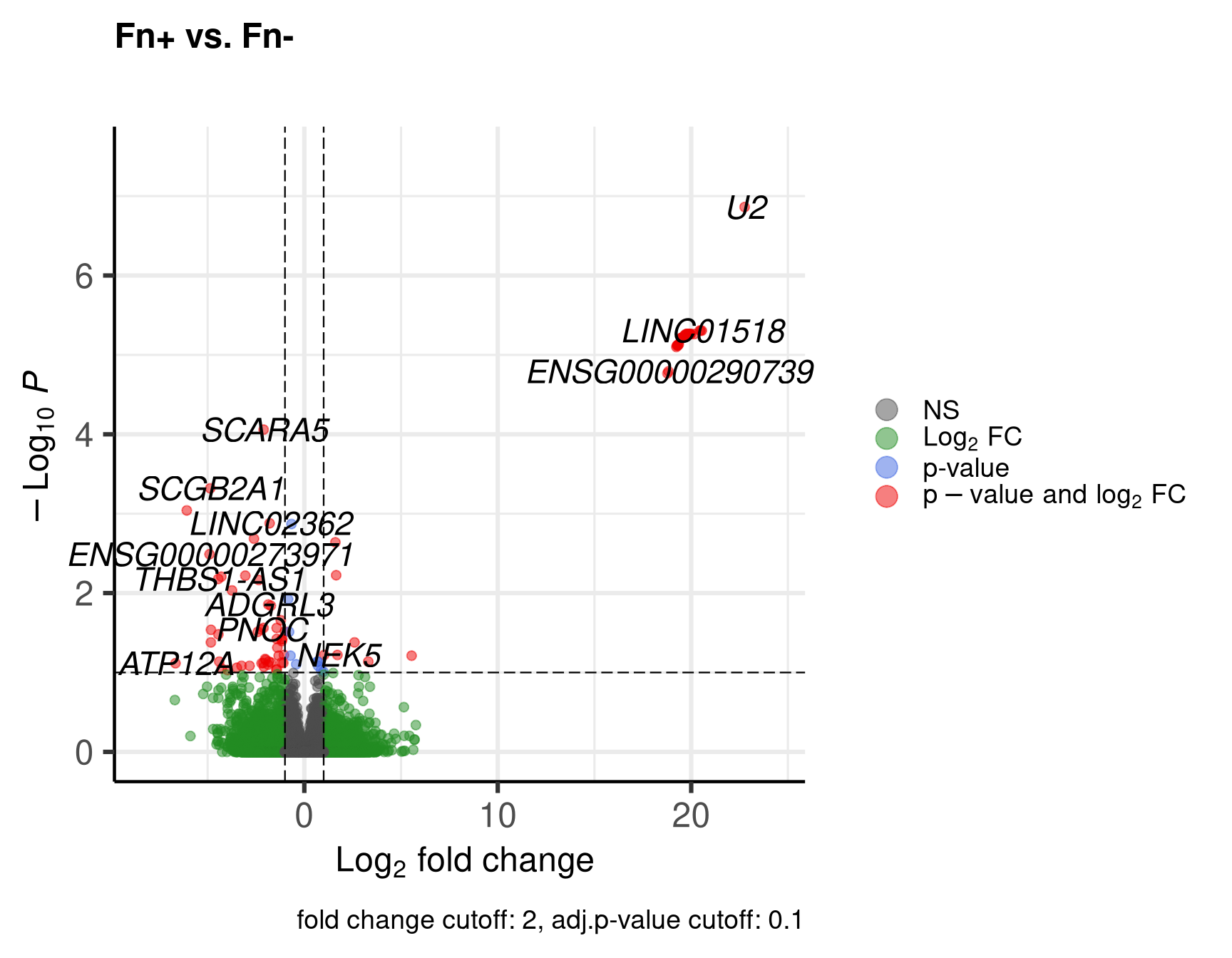
Supplementary Figure S6. Volcano plot displaying the differential gene expression analysis results. Significantly regulated genes (adjusted p-value < 0.1 and |log2FoldChange| >1) are shown in red with top regulated genes labeled.

References

# 1. Castellarin M, Warren RL, Freeman JD, Dreolini L, Krzywinski M, Strauss J, Barnes R, Watson P, Allen-Vercoe E, Moore RA. Fusobacterium nucleatum infection is prevalent in human colorectal carcinoma. Genome research. 2012;22(2):299-306.

# 2. Jo J, Choi S, Oh J, Lee S-G, Choi SY, Kim KK, Park C. Conventionally used reference genes are not outstanding for normalization of gene expression in human cancer research. BMC Bioinformatics. 2019;20(10):245.

# Supplementary Figures and Tables

For more information on Supplementary Material and for details on the different file types accepted, please see [here](https://www.frontiersin.org/guidelines/author-guidelines#supplementary-material).

## Supplementary Tables

| Target | Orientation | Sequence (5´ 🡪 3´) | Source |
| --- | --- | --- | --- |
| NusG gene (Fn ATCC25586) | Forward | CAACCATTACTTTAACTC TACCATGTTCA | Castellarin et al., 2012 [1] |
|  | Reverse | GTTGACTTTACAGAAGGA GATTATGTAAAAATC |  |
| PCBP1 (human) – for gDNA | Forward | TGATCATCGACAAGCTGGAG | Jo et al., 2019 [2] |
|  | Reverse | TCTTTGATCTTACACCCGCC |  |
| RPLp0 (human) – for cDNA | Forward | CCTGCGTGGCAATCCCTGAC |  |
|  | Reverse | GCCCACATTGTCTGCTCCCAC |  |

Supplementary Table S1. Primers used for qPCR of gDNA (colon cancer cohort) or cDNA (rectal cancer cohort).

| **Target** | **Clone** | **Metal** | **Incubation Time** | **Temp** | **Dilution** |
| --- | --- | --- | --- | --- | --- |
| CD8a | D8A8Y | 146 Nd | 5h | RT | 50 |
| PD-1 | D4W2J | 160 Gd | 5h | RT | 50 |
| ICOS | D1K2T(tm) | 161 Dy | 5h | RT | 50 |
| CD204 | J5HTR3 | 164 Dy | 5h | RT | 50 |
| CD103 | EPR4166(2) | 168 Er | 5h | RT | 50 |
| Tbet | 4B10 | 170 Er | 5h | RT | 50 |
| CD19 | D4V4B | 172 Yb | 5h | RT | 50 |
| CD163 | D6U1J | 173 Yb | 5h | RT | 50 |
| TGFbeta | TB21 | 115In | 5h | RT | 100 |
| HLA-DR | TAL 1B5 | 141 Pr | 5h | RT | 100 |
| CD11b | D6X1N | 144 Nd | 5h | RT | 100 |
| Granzyme B | D6E9W | 150 Nd | 5h | RT | 100 |
| Cleaved caspase | 5A1E | 155 Gd | 5h | RT | 100 |
| CD39 | EPR20627 | 157 Gd | 5h | RT | 100 |
| VISTA | D1L2G(TM) | 158 Gd | 5h | RT | 100 |
| CD14 | D7A2T | 163 Dy | 5h | RT | 100 |
| CD56 | E7X9M | 167 Er | 5h | RT | 100 |
| CD7 | EPR4242 | 174 Yb | 5h | RT | 100 |
| CD11c | EP1347Y | 176 Yb | 5h | RT | 100 |
| TCRgd + 2nd Ab | H41 | 148 Nd | Indirect ON | 4C | 50 |
| CD4 +2nd AB | EPR6855 | 145 Nd | Indirect ON | RT | 100 |
| CD45 | D9M8I | 149 Sm | Overnight | 4C | 50 |
| CD3 | EP449E | 153 Eu | Overnight | 4C | 50 |
| PD-L1 | E1L3N(R) | 156 Gd | Overnight | 4C | 50 |
| FOXP3 | D608R | 159 Tb | Overnight | 4C | 50 |
| CD27 | EPR8569 | 175 Lu | Overnight | 4C | 50 |
| Vimentin | D21H3 | 194 Pt | Overnight | 4C | 50 |
| Keratin | C11 and AE1/AE3 | 198 Pt | Overnight | 4C | 50 |
| B catenin | D10A8 | 89Y | Overnight | 4C | 100 |
| CD20 | H1 | 142 Nd | Overnight | 4C | 100 |
| CD68 | D4B9C | 143 Nd | Overnight | 4C | 100 |
| CD31 | 89C2 | 147 Sm | Overnight | 4C | 100 |
| CD57 | HNK-1 / Leu-7 | 151 Eu | Overnight | 4C | 100 |
| Ki-67 | 8D5 | 152 Sm | Overnight | 4C | 100 |
| P16ink4a | D3W8G | 154 Sm | Overnight | 4C | 100 |
| IDO | D5J4E(TM) | 162 Dy | Overnight | 4C | 100 |
| CD45RO | UCHL1 | 165 Ho | Overnight | 4C | 100 |
| D2-40 | D2-40 | 166 Er | Overnight | 4C | 100 |
| CD38 | EPR4106 | 169 Tm | Overnight | 4C | 100 |
| CD15 | MC480 | 171 Yb | Overnight | 4C | 100 |
| Histone H3 | D1H2 | 209 | Overnight | 4C | 50 |

Supplementary Table S2. Antibody panel used for imaging mass cytometry on tissue microarrays.

| **Cell Type** | **Cell class** | **Antigen function** |
| --- | --- | --- |
| CD4+ Tcells | immune | sensing |
| Tregs | immune | NA |
| CD8+ Tcells | immune | sensing |
| CD130+ Tcells | immune | NA |
| Gamma-delta Tcells | immune | NA |
| Undefined Tcells | immune | NA |
| CD19+ Tcells | immune | presenting |
| B cells | immune | presenting |
| Innate lymphoid cells | immune | NA |
| Monocytes | immune | NA |
| Macrophages | immune | presenting |
| Dendritic cells | immune | presenting |
| Granulocytes | immune | NA |
| Myeloid | immune | NA |
| Immune | immune | NA |
| Fibroblasts | fibroblasts | NA |
| tumorcells | tumorcell | presenting |
| Endothelial | endothelialcell | NA |

Supplementary table S3. Overview of the different cell types examined in the mixing scores. NA = not applicable.

**
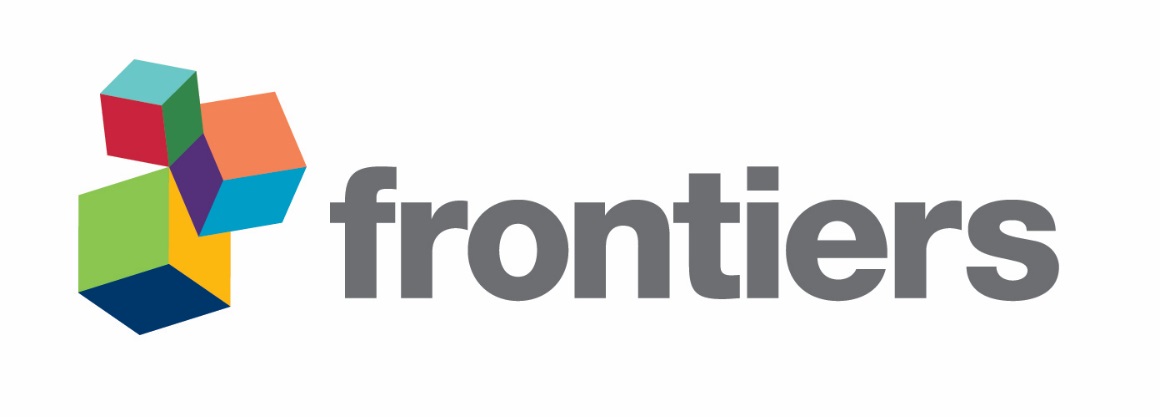
**
